# Effect of tyrosine kinase inhibitors, imatinib and nilotinib, in murine lipopolysaccharide-induced acute lung injury during neutropenia recovery

**DOI:** 10.1186/cc12786

**Published:** 2013-06-20

**Authors:** In Kyoung Kim, Chin Kook Rhee, Chang Dong Yeo, Hyeon Hui Kang, Dong Gun Lee, Sang Haak Lee, Jin Woo Kim

**Affiliations:** 1 Division of Pulmonary and Critical Care Medicine, Department of Internal Medicine, School of Medicine, The Catholic University of Korea, Seoul, Korea; 2 Division of Infectious Diseases, Department of Internal Medicine, School of Medicine, The Catholic University of Korea, Seoul, Korea

**Keywords:** Acute lung injury, neutropenia recovery, imatinib, nilotinib, platelet-derived growth factor receptor (PDGFR)

## Abstract

**Introduction:**

Neutrophil recovery has been implicated in deterioration of oxygenation and exacerbation of preexisting acute lung injury (ALI). The aim of this study was to investigate whether imatinib or nilotinib was effective on lipopolysaccharide (LPS)-induced ALI during neutropenia recovery in mice.

**Methods:**

Mice were rendered neutropenic with cyclophosphamide prior to the intratracheal instillation of LPS. Imatinib or nilotinib was administrated by oral gavage during neutropenia recovery. In order to study the effects of drugs, mice were killed on day 5 and blood, bronchoalveolar lavage (BAL) fluid and lung tissue samples were obtained. The lung wet/dry weight ratio and protein levels in the BAL fluid or lung tissue were determined.

**Results:**

Treatment with imatinib or nilotinib significantly attenuated the LPS-induced pulmonary edema, and this result was supported by the histopathological examination. The concentrations of tumor necrosis factor-α, interleukin (IL)-1β, IL-6 and myeloperoxidase in BAL fluid were significantly inhibited by imatinib or nilotinib in mice of ALI during neutropenia recovery. The mRNA expressions of platelet-derived growth factor receptor-β and c-KIT in imatinib or nilotinib group were significantly lower than LPS group.

**Conclusions:**

Our data indicated that imatinib or nilotinib effectively attenuated LPS-induced ALI during neutropenia recovery. These results provide evidence for the therapeutic potential of imatinib and nilotinib in ALI during neutropenia recovery.

## Introduction

Acute lung injury (ALI) and acute respiratory failure are the major cause of morbidity and the major reason for ICU admission in cancer patients [1-4]. Neutropenia, characterized by low count of neutrophils, which have a critical role in the pathophysiology of acute respiratory distress syndrome (ARDS) and ALI, is mostly a commonly expected event in the numerous cancer patients who are administered chemotherapy [5,6]. Neutropenia recovery may be related to an increased risk of deteriorating oxygenation and may exacerbate pre-existing ALI associated with infectious or noninfectious causes [7-11].

Most clinical studies have focused on the significant role of ALI before neutropenia recovery to detect confounding factors affecting the recovery. However, experimental studies to prevent or attenuate factors for ALI/ARDS after neutropenia recovery have been lacking to date, although ARDS has been widely reported during neutropenia recovery.

Lipopolysaccharide (LPS), a component of gram-negative bacterial endotoxin, is recognized as the main component causing ALI. It has been shown that ALI due to LPS instillation results in an increase in the numbers of total cells and neutrophils, as well as various proimflammatory cytokines such as TNF-α, IL-1β and IL-6 in bronchoalveolar lavage (BAL) fluid, and increased protein leakage, pulmonary elastance and resistance. There is also recent clinical evidence that increased TNF-α, IL-1β and IL-6 levels are associated with poor patient outcome in ALI.

Imatinib and nilotinib (Novartis Pharmaceuticals) are protein tyrosine kinase inhibitors whose main targets include platelet-derived growth factor (PDGF) receptor (PDGFR), discoidin domain receptor, stem cell factor receptor (KIT), Abelson kinase (ABL) and the oncogenic breakpoint cluster region-Abelson kinase (BCR-ABL) that causes chronic myeloid leukemia [12]. Imatinib and its advancer, nilotinib, have been shown to possess other beneficial pharmacological effects such as anti-inflammatory activities and antifibrotic effects [13,14]. There is evidence that these agents specifically attenuate airway hyper-reactivity [15] and its ability to inhibit PDGFR tyrosine kinase [14]. In a recent case report, PDGF has been known to play a key role in acute lung injury [16]. However, whether imatinib and nilotinib could affect ALI during neutropenia recovery and ultimately improve the ALI is unknown.

We hypothesized that imatinib and nilotinib may inhibit the cytokine production involved in the development of ALI. Therefore, the aim of the present study was to evaluate whether imatinib or nilotinib was effective in LPS-induced ALI during neutropenia recovery in a mouse model and whether these agents suppress the production of proinflammatory cytokines.

## Materials and methods

### Animals and treatment

Female 5-week-old ICR mice, weighing 18 to 22 g (*n *= 10 per group), were purchased from Orient Bio Experimental Animal Center, Kyoungki, Korea. All animals were maintained in a pathogen-free environment and had access to food and water *ad libitum*. Mice were randomly allocated into four groups: (i) control; (ii) cyclophosphamide + LPS (2 μg/g, Sigma, St. Louis, MO, USA); (iii) cyclophosphamide + LPS + imatinib (100 mg/kg, twice a day); and (iv) cyclophosphamide + LPS + nilotinib (100 mg/kg, once a day). Neutropenia was induced in the animals by intraperitoneal injections of cyclophosphamide of 150 mg/kg on day -5 (before imatinib or nilotinib administration) and 100 mg/kg on day -2. Imatinib or nilotinib (provided by Novartis Pharmaceuticals, Basel, Switzerland) was administered by oral gavage on day 0 and continued until euthanasia. In the groups (ii), (iii), and (iv), mice were given LPS (2 μg/g) through intratracheal instillation on day 2. Mice were sacrificed on day 5.

We also performed two additional experiments. First, we added two groups (LPS and saline) and compared the degree of lung injury. In the LPS group, mice was given LPS (2 μg/g) through intratracheal instillation without treatment of cyclophosphamide. Instead of LPS, the saline group received the same amount of saline through intratracheal instillation without cyclophosphamide treatment. Second, we gave imatinib or nilotinib after, instead of before, LPS administration. We compared the effect of imatinib or nilotinib in the pre- and post-LPS groups.

The experiments were approved by the ethical committee on animal experiments of The Catholic University of Korea.

### Quantification of lung wet/dry weight ratio

Mice were sacrificed by CO_2 _asphyxiation. At the completion of the experiment, the upper lobe of the right lung was excised and immediately weighed to determine the wet/dry (W/D) weight ratio. It was then dried at 60°C for 72 hrs, and reweighed. The ratio of W/D weight was used to quantify lung water content [17].

### Lung histopathology

After sacrifice, the lungs were inflated, fixed in 4% paraformaldehyde for 24 hrs, and then embedded in paraffin wax. Sections were cut at 4-μm thickness using a microtome, and stained with H&E using standard techniques for histological changes.

### Bronchoalveolar lavage

The trachea was cannulated with a small catheter, and BAL fluid was collected by washing the lungs with 0.8 ml of ice-cold sterile PBS. Total cell counts in BAL fluid were measured using a hemocytometer. Total leukocyte counts were obtained by light microscopic evaluation and the percentages of BAL fluid macrophages, neutrophils, eosinophils and lymphocytes were obtained by counting 500 leukocytes on randomly selected portions. BAL fluid was analyzed by cytospin stained with Diff Quik (Sysmax, Tokyo, Japan). The supernatant of BAL fluid was aliquoted and frozen at -80°C until further analysis.

### Myeloperoxidase activity assay

Myeloperoxidase (MPO) concentration in BAL fluid was determined using the Mouse MPO ELISA kit (Uscn Life Science, Wuhan, China) according to the manufacturer's instructions. Briefly, BAL fluid was centrifuged at 13,000 rpm for 10 minutes at 4°C and the supernatant discarded. The leukocyte pellet was suspended again in extraction buffer. The sensitivity of the assay was 0.78 ng/ml. Optical density was measured at 450 nm by use of a microplate reader.

### Enzyme-linked immunosorbent assay

The concentrations of TNF-α, IL-6 and IL-1β in BAL fluids were measured using an ELISA kit (R&D Systems, Minneapolis, MN, USA). The sensitivities of the assay were 15.6 pg/ml, 3 pg/ml and 7 pg/ml, respectively. The protocol was performed according to the manufacturer's instructions.

### Western blot

Separated lung tissues were homogenized in radio immunoprecipitation assay (RIPA) cell lysis buffer (150 mM NaCl, 1% triton X-100, 1% sodium deoxycholate, 0.1% SDS, 50 mM Tris-HCl, pH 7.5, and 2 mM ethylenediaminetetraacetic acid (EDTA)) containing a mixture of protease inhibitors (GenDEPOT, CA, USA) and then centrifuged at 13,000 rpm for 15 minutes at 4°C, followed by collection and storage of supernatant at -80°C. Protein samples were separated by 8% sodium dodecyl sulfate polyacrylamide gel electrophoresis and transferred to Immobilon-P (Millipore) polyvinylidene difluoride membranes. The membrane was blocked with 5% skimmed milk (Difco/Becton Dickinson, Atlanta, GA, USA) for 2 hrs at room temperature, followed by incubation with a 1:200 dilution of anti-PDGFR-β (Santa Cruz Biotechnology, Santa Cruz, CA, USA) or a 1:1,000 dilution anti-p-PDGFR-β antibody (Cell Signaling, Danvers, MA, USA) overnight at 4°C. After incubation, the blot was washed with Tris-buffered saline containing 0.1 % Tween 20 (TBS-T) for 30 minutes and incubated with a 1:2,000 dilution of goat anti-rabbit secondary antibody (Santa Cruz Biotechnology, Santa Cruz, CA, USA) for 2 hrs at room temperature. The blot was then washed with TBS-T for 1 hr, developed using the ECL Western Blotting Analysis System, and exposed to film.

### Real-time polymerase chain reaction

Total RNA was extracted from lung tissue using the TRIzol reagent™ (Invitrogen, Carlsbad, CA, USA) according to the manufacturer's recommendations. After extraction and quantification of total RNA, real-time PCR reactions were performed using a QuantiTect SYBR Green RT-PCR kit (Qiagen, Valencia, CA, USA). Each PCR was carried out in a final volume of 25 μl (100 ng cDNA, 1 μmol/l of primers, 2× SYBR RT-PCR Master Mix, RNase-free water). The PCR conditions were 95°C (10 minutes), followed by 40 cycles at 95°C (15 sec), 60°C (1 minute), and the standard denaturation curve. Primer sequences used were: PDGFR-β [NCBI:NM001146268.1], forward 5'- TGGGCTTCAGCTACCAAGTG-3', reverse 5'- AAGGTGCTGCCTTTGGAGAT-3'; β-actin (housekeeping control, [NCBI:NM007393.3]), forward 5'-ACAGGAAGTCCCTTGCCATC-3', reverse 5'-AGGGAGACCAAAAGCCTTCA-3'. Gene expression was quantified using standard curves for the respective cDNA products. To normalize the content of cDNA samples, the comparative threshold (CT) cycle method, consisting of the normalization of the number of target gene copies versus the housekeeping gene β-actin, was used. The changes in target signal were expressed as:

$$\text{Δ}{\text{C}}_{\text{T}}=\text{Δ}{\text{C}}_{\text{T,treatment}}-\text{Δ}{\text{C}}_{\text{T,control}}$$

Relative changes were calculated as 2^-ΔΔCT^.

### Statistical analysis

The data were analyzed by one-way analysis of variance (ANOVA) followed by Dunnett's multiple range test, using Graph-Pad Prism version 5.00 for Windows (GraphPad Software, San Diego, CA, USA). All data are expressed as means ± SD, and a *P-*value <0.05 was accepted as statistically significant. SPSS for Windows was used for the statistical tests.

## Results

### Effect of cyclophosphamide on neutrophils

**Figure 1:**
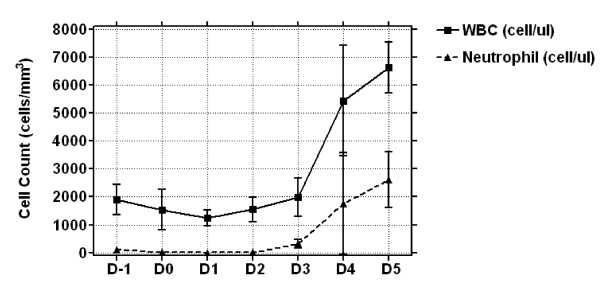
**Effect of cyclophosphamide in mice**. All mice received 150 mg/kg cyclophosphamide by intraperitoneal injection on day (D) -5 before they became neutropenic and 100 mg/kg on day -2 before administration of imatinib or nilotinib. Neutrophils were measured in the peripheral blood every day to monitor the cytotoxic effect of cyclophosphamide until mice were sacrificed. WBC, white blood cell.

The cytotoxic effect of cyclophosphamide in mice was monitored by neutrophil counts. In mice given cyclophosphamide, neutrophils in the peripheral blood reached a minimum at day 1 and recovered at day 5 (Figure 1).

### Effect of imatinib or nilotinib on lung histopathology

**Figure 2:**
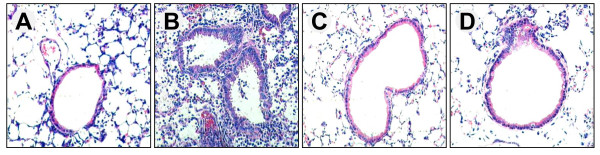
**Effect of imatinib or nilotinib on the histopathological changes in the lung in mice lipopolysaccharide-induced acute lung inury**. Representative images of H&E-stained lung sections from four experimental groups: (**A**) control group; the lung structure is normal, (**B**) cyclophosphamide (C) + lipopolysaccharide (LPS) group; LPS induced acute lung damage with interstitial edema, hemorrhage, thickening of the alveolar wall and infiltration of inflammatory cells into the interstitium and alveolar spaces, (**C**) C + LPS + imatinib (I) group, (**D**) C + LPS + nilotinib (N) group; lung injury was attenuated by treatment with imatinib or nilotinib.

To evaluate the effect of imatinib or nilotinib in the histopathological changes in the lung in LPS-induced ALI, lung tissues were assessed after the administration of LPS during neutropenia recovery with or without treatment. We observed marked acute alveolar damage, acute inflammation and interstitial edema in LPS-induced ALI during neutropenia recovery (Figure 2B), compared with the control group (Figure 2A). In the group pretreated with imatinib or nilotinib before LPS, administration of drugs effectively reduced the inflammatory changes in the lung (Figure 2C and 2D).

### Effect of imatinib or nilotinib on pulmonary edema

**Figure 3:**
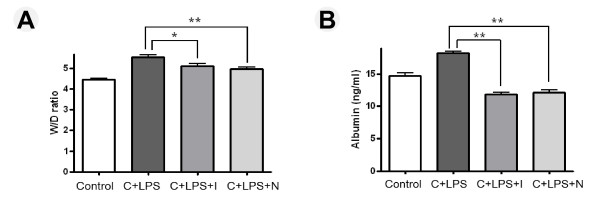
**Effect of imatinib or nilotinib on lung edema in mice with lipoplysaccharide-induced acute lung injury**. After lipopolysaccharide (LPS) administration, treatment with imatinib or nilotinib markedly decreased (**A**) lung wet/dry (W/D) weight ratio and (**B**) albumin. The lower lobe of the right lung was removed and weighed (wet weight), dried at 60°C for 72 hrs, and weighed again (dry weight). The W/D ratio was used as an indicator of lung edema formation. C, cyclophosphamide; I, imatinib; N, nilotinib. Asterisks indicates significant differences to the C + LPS group (* *P *<0.05, ** *P *<0.01).

Pulmonary edema formation, indicative of pulmonary vascular leakage, happens with an increase in the lung W/D ratio. To investigate the effect of imatinib or nilotinib on LPS-induced lung edema, the lung W/D ratios and the concentration of albumin in BAL fluids were measured. As shown in Figure 3A, we found that there was an obvious increase in W/D ratio in the group that had LPS administration during neutropenia recovery, compared with the control group. However, administration of imatinib or nilotinib prior to LPS significantly reduced the lung W/D ratios. The level of albumin in BAL fluid was increased in mice challenged with LPS during neutropenia recovery compared with control mice, whereas treatment with imatinib or nilotinib prior to LPS significantly attenuated the albumin level (Figure 3B).

### Effect of imatinib or nilotinib on inflammatory cells

To investigate the protective effect of imatinib or nilotinib on LPS-induced ALI during neutropenia recovery, we detected the total inflammatory cell counts in BAL fluid from mice treated with LPS with or without imatinib or nilotinib. As shown in table 1, inflammatory cells and neutrophils in the BAL fluid were significantly increased after administration of LPS during neutropenia recovery (*P <*0.01), whereas we observed that administration of imatinib or nilotinib significantly reduced the number of total cells and neutrophils compared with the LPS group (*P <*0.01).

**Table 1:** Results of bronchiolar lavage fluid analysis (10^4^/ml) in the four groups

	Control	C + LPS	C + LPS + I	C + LPS + N
Total cells	6.00 ± 2.35	80.60 ± 16.30**	36.20 ± 7.26^##^	43.40 ± 6.91^##^
Macrophages	3.67 ± 2.15	4.18 ± 0.68	5.63 ± 3.20	3.49 ± 4.88
Lymphocytes	0.00 ± 0.00	0.34 ± 0.56	0.00 ± 0.00	0.00 ± 0.00
Neutrophils	0.00 ± 0.00	71.46 ± 16.09**	26.38 ± 2.85^##^	14.46 ± 20.24^##^
Eosinophils	0.34 ± 0.21	4.64 ± 3.95	4.20 ± 4.81	2.04 ± 2.83

Results are presented as mean ± SD. LPS, lipopolysaccharide; C, cyclophosphamide; I, imatinib; N, nilotinib. **P <0.01 for control versus C + LPS; ^##^*P *<0.01 for C + LPS versus C + LPS + I or N.

### Effect of imatinib or nilotinib on inflammatory cytokines and MPO

**Figure 4:**
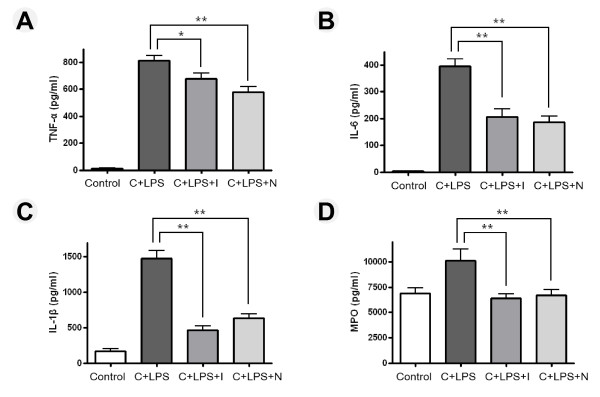
**Effect of imatinib or nilotinib on the protein level of inflammatory cytokines and myeloperoxidase in mice with lipoplysaccharide-induced acute lung injury**. The levels of (**A**) TNF-α, (**B**) IL-6, (**C**) IL-1β and (**D**) myeloperoxidase (MPO) were assayed in bronchiolar lavage (BAL) fluid and the sensitivities of the assay were 15.6 pg/ml, 3 pg/ml, 7 pg/ml and 0.78 ng/ml, respectively. Intratracheal administration of lipopolysaccharide (LPS) induced increased levels of TNF- α, IL-6, IL-1β and MPO. Compared with the cyclophosphamide (C) + LPS group, the level of inflammatory cytokines and MPO was significantly lower in the imatinib or nilotinib group. I, imatinib; N, nilotinib. Asterisks indicates significant differences to the C + LPS group (*P <0.05, **P <0.01).

To assess the anti-inflammatory effect of imatinib or nilotinib, we analyzed the proinflammatory cytokines, including TNF-α, IL-6, IL-1ß, and MPO in BAL fluid. We found that the concentrations of TNF-α, IL-6, IL-1ß, and MPO were dramatically increased in BAL fluid after LPS administration during neutropenia recovery. The concentrations of TNF-α, IL-6 and IL-1ß in response to LPS were 812.16 ± 84.88 pg/ml, 394.19 ± 67.00 pg/ml and 1,476.81 ± 268.95 pg/ml, respectively, indicating approximately 58.2-, 109.0- and 8.8-fold increases compared with control group. In contrast, administration of imatinib or nilotinib after LPS challenge effectively decreased the levels of TNF-α, IL-6, IL-1ß and MPO (Figure 4).

### Effect of imatinib or nilotinib on activation of PDGFR-ß

**Figure 5:**
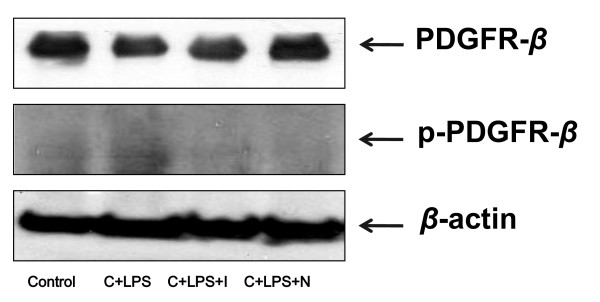
**Effect of imatinib or nilotinib on activation of p-platelet-derived growth factor receptor-ß in mice with lipoplysaccharide-induced acute lung injury**. Lung tissues were homogenized and the extracted protein samples were separated by SDS-PAGE using 8% PAGE gel, followed by transfer to 0.45 μm polyvinylidene fluoride membranes. The membranes were blocked with 5% skimmed milk and exposed overnight at 4°C to specific anti-platelet-derived growth factor receptor-ß (PDGFR-β) and anti-phospho-PDGFR-β antibodies. LPS, lipopolysaccharide; C, cyclophosphamide; I, imatinib; N, nilotinib.

Western blot analysis of the phosphorylation of PDGFR-ß revealed significant upregulation in the group with LPS during neutropenia recovery, whereas the group treated with imatinib or nilotinib there was a reduction toward control levels (Figure 5). There was no significant difference in the expression of non-phosphorylated PDGFR-ß in mice exposed to LPS during neutropenia recovery compared with the control group.

### Effect of imatinib or nilotinib on mRNA expression of PDGFR-ß

**Figure 6:**
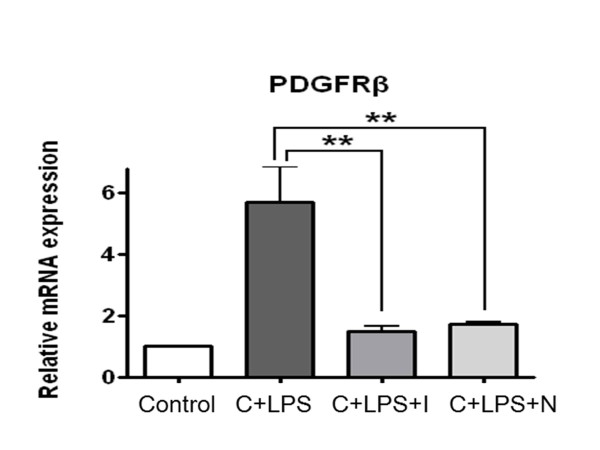
**Effect of imatinib or nilotinib treatment on mRNA expression of platelet-derived growth factor receptor in mice with lipoplysaccharide-induced acute lung injury**. The mRNA expression of platelet-derived growth factor receptor-ß (PDGFR) was measured in lung tissues. In real-time PCR analysis, to normalize the content of cDNA samples, the comparative threshold (CT) cycle method, consisting of the normalization of the number of target gene copies versus the housekeeping gene β-actin, was used. LPS, lipopolysaccharide; C, cyclophosphamide; I, imatinib; N, nilotinib. Asterisks indicate significant differences from the C + LPS group (** *P *<0.01).

Real-time PCR was used to analyze the effect of imatinib or nilotinib on the mRNA expression of PDGFR-ß in lung tissue after the LPS challenge. As shown in Figure 6, the administration of LPS during neutropenia recovery induced a significant increase in the mRNA expression of PDGFR-ß as compared to the control group. Imatinib or nilotinib significantly reduced LPS-induced PDGFR-ß expression (Figure 6, *P <*0.01).

### Comparison of degree of lung injury

**Figure 7:**
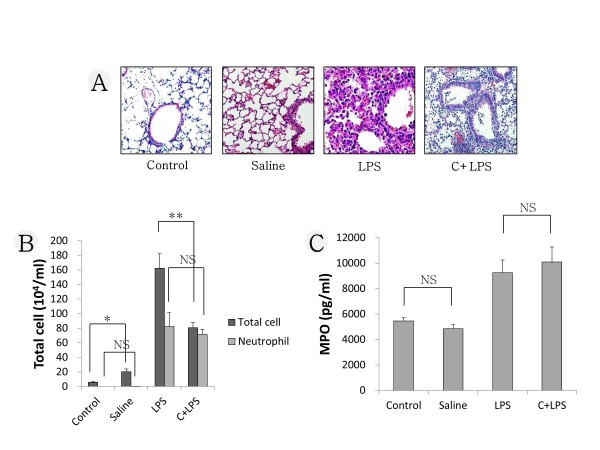
**Comparison of degree of lung injury**. (**A**) Representative images of H&E-stained lung sections from four experimental groups. (**B**) Number of inflammatory cells in bronchoalveolar lavage (BAL) fluid. (**C**) Result of myeloperoxidase (MPO) analysis. LPS, lipopolysaccharide; C, cyclophosphamide; I, imatinib; N, nilotinib. *P <0.05; **P <0.01; NS, non-significant.

We compared the degree of lung injury in the LPS group and the cyclophosphamide + LPS group. We also compared the control and the saline group. In the histopathology, there was little inflammation in the control or the saline group. Acute lung damage with interstitial edema, hemorrhage, thickening of the alveolar wall and infiltration of inflammatory cells into the interstitium and alveolar spaces were observed in both the LPS and the cyclophosphamide + LPS group (Figure 7A). In BAL cell count analysis, there was a significant difference between the control and the saline group in the number of total cells (*P *<0.05). However, the neutrophil count did not differ significantly between the control and the saline group. There was also significant difference between the LPS group and the cyclophosphamide + LPS group in the number of total cells (*P *<0.01). However, the neutrophil count did not differ significantly in the LPS group and the cyclophosphamide + LPS group (Figure 7B). In the analysis of MPO, there was no significant difference between the control and the saline group. There was also no significant difference between the LPS group and the cyclophosphamide + LPS group (Figure 7C).

### Post-treatment effect of imatinib or nilotinib

**Figure 8:**
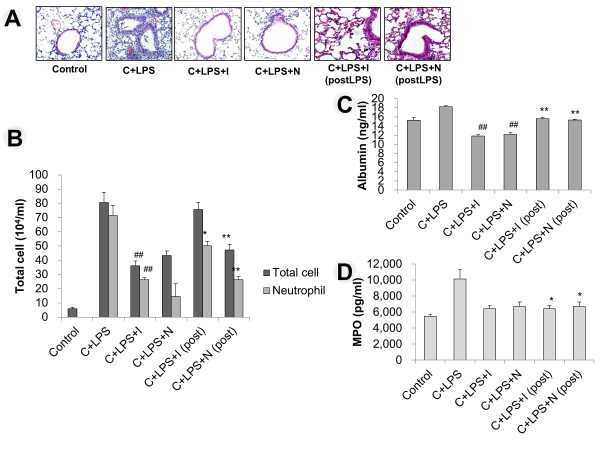
**Effect of post-treatment with imatinib or nilotinib**. (**A**) Representative images of H&E-stained lung sections from six experimental groups. (**B**) Number of inflammatory cells in bronchoalveolar lavage (BAL) fluid. (**C**) Albumin level in BAL fluid. (**D**) Myeloperoxidase (MPO) analysis. LPS, lipopolysaccharide; C, cyclophosphamide; I, imatinib; N, nilotinib. *P <0.05 and **P <0.01 for C + LPS versus C + LPS + I/N (post); ^#^*P *<0.05 and ^##^*P *<0.01 for C + LPS + I/N versus C + LPS + I/N (post). Post, post-induction of LPS.

In the histopathology, imatinib or nilotinib given pre- or post-induction of lung injury attenuated lung injury compared with cyclophosphamide + LPS (Figure 8A). Compared with the cyclophosphamide + LPS group, the total cell count did not differ significantly in the group that received imatinib post treatment. However, the neutrophil count differed significantly in the group receiving imatinib post treatment (*P *<0.05). Compared with cyclophosphamide + LPS group, both total cell and neutrophil count were significantly lower in post-treatment of nilotinib group (*P *<0.01). The total cell and neutrophil counts were significantly higher in post-treatment imatinib group compared with the pre-treatment group (*P *<0.01). However, there was no significant difference between the pre- and post-treatment nilotinib group in the numbers of total cells and neutrophils (Figure 8B). Compared with the cyclophosphamide + LPS group, the albumin level was significantly lower in the imatinib and nilotinib post-treatment groups (*P *<0.01). Compared with imatinib and nilotinib post-treatment groups, the albumin level was also significantly lower in the imatinib and nilotinib pre-treatment groups (Figure 8C, P <0.01). Compared with the cyclophosphamide + LPS group, MPO was significantly lower in the imatinib and nilotinib post-treatment groups (*P *<0.05). There was no significant difference in MPO between the imatinib or nilotinib pre- and post-treatment groups (Figure 8D).

## Discussion

In this study, we observed the pretreatment effect of imatinib and nilotinib in LPS-induced lung injury in the neutropenic mouse model. Although many cases of ALI/ARDS during neutropenia recovery have been reported [18], risk factors or marked therapeutic agents in patients experiencing neutropenia recovery have not previously been studied.

Chemotherapy is administered to the majority of cancer patients, with neutropenia being a frequent adverse event. In patients, pulmonary infiltrates develop during neutropenia and considerably worsen upon neutropenia recovery [10]. A few case reports have been published showing that neutropenia recovery carries a risk of acute respiratory failure such as ALI and ARDS [19,20]. Azoulay *et al. *[20] showed that cancer patients recovering from neutropenia were frequently admitted for acute respiratory failure and that one third of patients experienced ARDS during neutropenia recovery. During neutropenia recovery, the accumulation of activated neutrophils in the pulmonary vasculature generally occurred [6]. Activated neutrophils that quickly accumulate in the pulmonary parenchyma following endotoxin administration [21] or hypovolemic shock [22] have a major role in the development of inflammatory response in ALI [23]. Inflammatory cells, including neutrophils and lymphocytes, in BAL fluid have a principal role in the pathogenesis of ALI [24].

PDGF, one of the growth factors whose receptors are targeted by imatinib and nilotinib, plays a key role in the pathogenesis of lung diseases including pulmonary fibrosis, ALI and ARDS [17,25]. PDGF is a chemotactic factor for monocytes and granulocytes during inflammation and overexpression of PDGF can induce inflammatory injury [26]. Previous studies suggest that PDGF may be an important factor in ALI. Snyder *et al. *[27] showed that the concentration of PDGF is significantly higher in patients with ALI than in normal or control patients. Budinger *et al. *[28] showed that there was a significant negative correlation between active TGF-β1(stimulator of PDGF) levels and ventilator-free days and ICU-free days. In addition to these clinical data, Walsh *et al. *[29] showed that PDGF plays a crucial role in the tissue repair processes of bleomycin-induced lung injury in a rat model and leads to fibroblast proliferation and chemotaxis. There are also many reports that on PDGF as a therapeutic target in ALI. In a previous study, we showed that imatinib and nilotinib attenuated bleomycin-induced ALI by blocking the PDGF pathway [30]. Zhao *et al. *[31] observed therapeutic effects of bone marrow-derived mesenchymal stem cells in lung injury and this effect was accompanied by a decreased in PDGF. Yi and colleagues [32] showed that keratinocyte growth factor can attenuate bleomycin-induced lung injury by decreasing the expression of PDGF.

Recent evidence showed that imatinib and nilotinib have various effects in airway hyper-reactivity and inflammatory responses [30,33-35] as they specifically inhibit PDGFR tyrosine kinase [14,16,17]. In this study, we found that treatment of imatinib or nilotinib significantly decreased the phosphorylation of PDGFR-ß. We also confirmed that imatinib or nilotinib could decrease the mRNA expression of PDGFR-ß. These previous reports together with our results suggest that inhibition of PDGF/PDGFR may be a potential approach to prevent lung injury.

We have observed that imatinib and nilotinib have an anti-inflammatory effect. This beneficial effect could contribute to attenuation of lung injury. In BAL fluid analysis, total cells and neutrophils in the BAL fluid were significantly decreased by treatment with imatinib or nilotinib in mice with LPS-induced ALI during neutropenia recovery. Similarly, MPO activity, which indicates neutrophil and macrophage leakage, was significantly increased in BAL fluid after the administration of LPS. These results confirm that a potent protective effect of imatinib or nilotinib on LPS-induced ALI during neutropenia recovery is related to an attenuation of lung inflammation and tissue neutrophilia. The anti-inflammatory effect of imatinib and nilotinib is consistent with previous reports. Miyachi *et al. *[36] and Eklund *et al. *[37,38] reported that imatinib has an anti-inflammatory effect and this drug can be used for the treatment of rheumatoid arthritis. In animal studies previous experiments consistently showed that imatinib and nilotinib decreased total cells and/or neutrophils in BAL fluid [15,30,33].

It has been shown that the early release of several inflammatory and chemotactic cytokines, such as TNF-α, IL-6 and IL-1ß, enlarge and facilitate inflammatory responses in ALI [39,40]. These are the primary multifunctional cytokines produced from inflammatory cells that exacerbate the extent of lung injury [41]. As in previous studies [42,43], our results showed that the expressions of TNF-α, IL-6 and IL-1ß were significantly increased in BAL fluid after LPS administration as compared to control group, whereas administration of imatinib or nilotinib significantly downregulated the expression of these cytokines as compared to the LPS group.

## Conclusions

In the present study, we found that administration of imatinib or nilotinib effectively attenuated the LPS-induced ALI during neutropenia recovery in mice. These effects of imatinib or nilotinib were associated with the inhibition of infiltration of inflammatory cells into the lung and reduction of the activation of inflammatory cytokines. Moreover, the beneficial effects of imatinib and nilotinib were closely connected with inhibition of the PDGF pathways, because the PDGF signaling pathway has been involved in developing organ fibrosis and inflammation [44]. However, the accurate intracellular mechanism of the effects of imatinib and nilotinib in mice with LPS-induced ALI still remains to be elucidated. Furthermore, it is necessary to confirm these results in more clinically relevant models.

## Key messages

• Pretreatment with imatinib or nilotinib before LPS exposure during neutropenia recovery, effectively prevented the degree of pulmonary edema and reduced the inflammatory changes in lung tissues.

• Administration of imatinib or nilotinib before instillation of LPS during neutropenia recovery significantly downregulated several inflammatory and chemotactic cytokines, such as TNF-α, IL-6 and IL-1ß.

• Pretreatment with imatinib or nilotinib before instillation of LPS during neutropenia recovery, significantly decreased the phosphorylation of PDGFR-ß as well as the mRNA expression.

## Abbreviations

ABL: Abelson kinase; ALI: acute lung injury; ANOVA: analysis of variance; ARDS: acute respiratory distress syndrome; BAL: bronchoalveolar lavage; BCR-ABL: breakpoint cluster region-Abelson kinase; CT: comparative threshold; EDTA: ethylenediaminetetraacetic acid; ELISA: enzyme-linked immunosorbent assay; H&E: hematoxylin and eosin; IL: interleukin; KIT: stem cell factor receptor; LPS: lipopolysaccharide; MPO: myeloperoxidase; PBS: phosphate-buffered saline; PCR: polymerase chain reaction;PDGF: platelet-derived growth factor; PDGFR: platelet-derived growth factor receptor; RIPA: radio immunoprecipitation assay; TBS-T: Tris-buffered saline containing 0.1 % Tween 20; TNF: tumor necrosis factor; W/D: wet/dry.

## Competing interests

The authors declare that they have no competing interests.

## Authors' contributions

CKR and JWK designed this study. IKK participated in the animal experiment. IKK, CKR and JWK analyzed the data. DGL contributed to the neutropenia model. IKK, CKR, CDY, HHK, SHL and JWK drafted the manuscript. All authors read and approved the final manuscript.
